# Concealed primary aortic sarcoma induced hypertensive encephalopathy resulting from a thoracic aortic occlusion: a case report

**DOI:** 10.1186/1749-8090-8-102

**Published:** 2013-04-18

**Authors:** Hyunmin Choi, Hee-Jeoung Yoon, Woo-Ik Jang, Chang-Young Kim, Joon-Hyung Doh

**Affiliations:** 1 Division of Cardiology, Gumdan Top General Hospital, Dangha-dong, Seo-gu, Incheon 404-310, South Korea; 2 Division of Thoracic and Cardiovascular Surgery, Inje University College of Medicine, Goyang-si, South Korea; 3 Division of Cardiology, Ilsan Paik Hospital, Inje University College of Medicine, Goyang-si, South Korea

**Keywords:** Aorta, Sarcoma, Hypertensive encephalopathy

## Abstract

Primary aortic sarcoma is a rare condition that is frequently associated with distal embolization. In addition, growth characteristics of primary aortic sarcoma lead to the narrowing of the involved aortic lumen. A 72-year-old Korean male with primary aortic sarcoma showed progressive unexplained blood pressure elevation that didn’t improve with additional antihypertensive drug therapy. Because follow-up measures were not taken, the patient ultimately developed hypertensive encephalopathy with concurrent embolic dissemination. Although we successfully performed open transcatheter embolectomy in both legs, the patient died because of multiple organ failure 3 days after surgery. Given the ominous prognosis for this condition, this case report highlights the fact that the value of early detection and prompt evaluation of altered vital signs should not be overemphasized. We describe a rare case of primary aortic sarcoma that showed hypertensive encephalopathy caused by thoracic aortic occlusion and also had embolic metastases to the lower extremities.

## Background

Primary aortic sarcoma (PAS) is a very rare and aggressive malignancy that has been frequently associated with embolic events associated with broken pieces of tumor. However, the condition of the surrounding tissue may lead to narrowing of the involved aortic lumen. If aortic luminal narrowing becomes significant, hypertensive encephalopathy may occur because of the backward pressure overload, as with coarctation of the aorta.

In this case report, we present a rare case of PAS that required open transcatheter embolectomy for the treatment of malperfusion due to metastatic emboli to the lower extremities, with concurrent hypertensive encephalopathy caused by thoracic aortic occlusion.

## Case presentation

**Figure 1:**
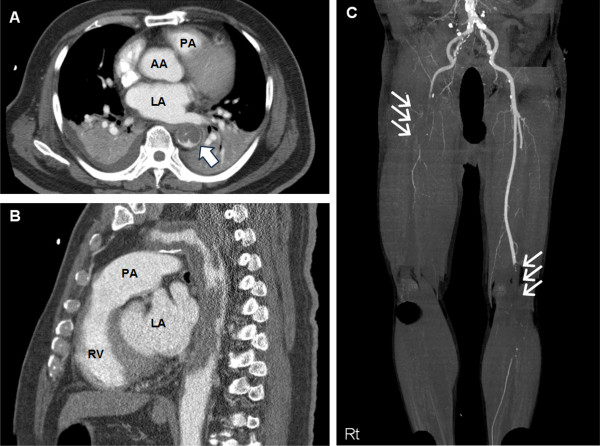
**Images of the patient’s 3DCT before surgery.** (**A**) Axial and (**B**) sagittal section 3DCT scans of the aorta show near total occlusion of the descending thoracic aorta that is blocked with diffusely distributed thrombus-like mass (single white arrow). (**C**) 3DCT scan of the lower extremities shows total occlusion of the right common iliac artery (right triple white arrows) and left superficial femoral artery (left triple white arrows). 3DCT: three-dimensional computed tomography; PA: pulmonary artery; AA: ascending aorta; LA: left atrium; RV: right ventricle.

A previously healthy 72-year-old Korean male was transferred to Ilsan Paik hospital because of restlessness and mental confusion for 2 hours. He had essential hypertension and type 2 diabetes since 25 years. Two months before these events, his local physician adjusted antihypertensive medications for unexpectedly uncontrollable hypertension despite full-dose combinations of 3 different antihypertensive drugs (angiotensin-2 receptor blockers, beta-blockers, and calcium channel blockers). However, the patient was managed medically without further evaluation of the uncontrollable hypertension. Immediately before experiencing mental confusion, he complained of severe headache and vomiting. Initial vital signs showed blood pressure of 220/130 mmHg, pulse rate 110 beats/min, respiration rate of 30 breaths/min, and weak pulses in the right superficial femoral and both dorsalis pedis arteries. Suddenly, his respirations and oxygen saturation level began worsening; therefore, we promptly performed endotracheal intubation. After endotracheal intubation, we suspected intracranial hemorrhage or acute aortic syndrome; therefore, we conducted emergency three-dimensional computed tomography (3DCT) scans of the brain, aorta, and lower extremities. The 3DCT scan of the brain showed no evidence of intracranial bleeding or infarct. However, the 3DCT of the aorta and lower extremities demonstrated that the descending thoracic aorta was almost totally occluded by intraluminal masses resembling mural thrombus (Figure 1A). Additionally, total occlusion of the right common iliac artery and left superficial femoral artery were noted in 3DCT scans of the lower extremities (Figure 1B). Both renal arteries were maintaining patency without any filling defect. Moreover, several enlarged peribronchial lymph nodes and large pleural effusions were also detected. The intraluminal masses resembling mural thrombus were characterized as having a homogenous and lobulated soft-tissue density. These characteristic findings strongly suggested a primary aortic neoplasm as opposed to mural thrombus. We attributed the patient’s symptoms to a descending thoracic aortic tumor, with hypertensive encephalopathy induced by backward blood pressure overload and secondary embolization of the lower extremities.

**Figure 2:**
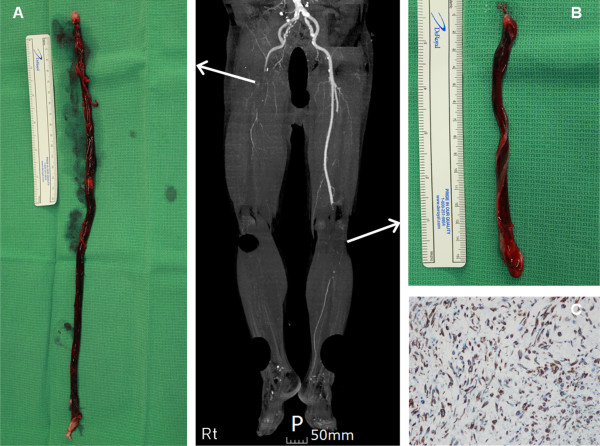
**Gross and microscopic findings of the embolic mass extracted from both lower extremities.** The masses extracted by open transcatheter embolectomy at (**A**) the right common iliac artery and (**B**) the left superficial femoral artery have a long rod consisting of gray soft tissue and red thrombus. (**C**) Immunohistochemical staining of the tumor cells confirms intimal-type PAS positive for vimentin. PAS: primary aortic sarcoma.

First, we planned to perform an open transcatheter embolectomy of the totally occluded right common iliac and left superficial femoral arteries as well as left axillofemoral arterial bypass surgery. Open surgical transcatheter embolectomy was done by using a Fogarty catheter via both superficial and deep femoral arteries to restore blood flow to both lower extremities. Two long rod-shaped masses (right mass, 373 × 18 mm; left mass, 157 × 17 mm) were extracted from the femoral arteriotomy sites (Figure 2A and B). Both embolic masses consisted of irregular gray soft tissue and red thrombus. The embolic mass was sent for pathological analysis. However, we left the thoracic aortic mass untouched, and 40 mg of enoxaparin sodium was administered subcutaneously because the patient’s vital signs were very unstable.

Dorsalis pedis arterial pulsations and vascular Doppler flow signals were both restored immediately after arteriotomy closure. However, 1 day after the operation, the patient’s vital signs and the degree of metabolic acidosis worsened. We tried to correct the patient’s vital signs and metabolic acidosis but he could not endure the treatment. Subsequently, the patient died of multiple organ failure, 3 days after open surgery. At post-mortem, the embolic mass was positive for vimentin and negative for CD 34 (Figure 2C) on immunohistochemical staining. This histopathologic report confirmed the diagnosis of intimal-type PAS.

## Discussion

PAS is a very rare tumor. Most cases of PAS more frequently may involve the descending thoracic or abdominal aorta than the aortic arch [1]. PAS have been divided into intimal and mural types. Of the 2 types, the intimal type is more aggressive and tends to spread extensively along the aortic lumen.

The initial presentation symptoms are diverse and nonspecific, varying according to the individual tumor’s growth and invasion patterns. The most common symptoms are ischemic embolic events that involve the lower extremities, intestinal, or renal arteries and which require emergency surgical treatment [2]. The intimal type of aortic sarcoma forms protruding intraluminal masses that can lead to embolic dissemination through the bloodstream [1].

Antemortem diagnosis of the aortic sarcoma is very difficult, because the presenting symptoms may be too vague to allow the physician to seize the opportunity for treatment [3]. Our case would have masked as ordinary hypertensive encephalopathy, if weak pulses in the bilateral lower extremities would not have been detected. Hypertensive encephalopathy could have been caused by tumor embolization of the renal arteries [4]; however, in our patient, both renal arteries were intact. Therefore, we propose that the mechanism of the hypertensive encephalopathy was narrowing of the involved thoracic aorta, which triggered the backward flow blood before the obstructed segment, resulting in elevated blood pressure.

In most of the reported cases, surgical treatment was performed despite this condition being advanced and incurable. In the absence of distant metastasis, radical surgical resection and graft interposition is the optimal treatment scenario. Although chemotherapy or radiation therapy can be utilized in advanced or metastatic conditions, there is little evidence of therapeutic benefit from chemotherapy or radiation therapy [5]. PAS in our case was diagnosed at a later stage; therefore, we were probably could not save our patient. This case underlines how an active approach to the patient who shows sudden uncontrolled blood pressure is needed for detection of any hidden disease. Although 3DCT or magnetic resonance imaging of the heart and aorta are generally available in many cardiac centers, not all physicians routinely recommend these modalities for evaluation of secondary hypertension. We recommend that careful investigation and accurate imaging modalities should be considered to search for the unexplained cause of sudden elevation of blood pressure in patients with primary hypertension.

## Conclusions

We reported a rare case of PAS that resulted in hypertensive encephalopathy due to an obstructed thoracic aorta. If an unexplained blood pressure elevation occurs in patients with primary hypertension despite active medical therapy, a multilateral evaluation for secondary disease should be performed. In this way, we may acquire important clues for the early detection and proper management of concealed diseases such as PAS.

## Consent

Written informed consent was obtained from the patient’s family for publication of this report and any accompanying images.

## Abbreviations

PAS: Primary aortic sarcoma; 3DCT: Three-dimensional computed tomography.

## Competing interests

The authors declare that they have no competing interests.

## Authors’ contributions

WJ and CK performed the surgical operations, HC and HY wrote the manuscript. JD revised the manuscript. All authors read and approved the final manuscript.
